# Albumin–Globulin Ratio Is an Independent Determinant of 28-Day Mortality in Patients with Critical Illness

**DOI:** 10.1155/2021/9965124

**Published:** 2021-08-25

**Authors:** Bin Liu, Kun Xiao, Peng Yan, Tianyu Sun, Jiang Wang, Fei Xie, Guoxin Mo, Lixin Xie

**Affiliations:** ^1^College of Pulmonary and Critical Care Medicine, Chinese PLA General Hospital, China; ^2^Medical School of Chinese PLA, China

## Abstract

**Background:**

Critical illness in the intensive care unit (ICU) has been a global health priority. Systemic nutritional status has turned out to be related to the prognosis of critically ill patients. The albumin-globulin ratio (AGR) has been reported to be a novel prognostic factor of many diseases. This study is aimed at investigating whether the AGR could predict the mortality risk in critically ill patients.

**Methods:**

We enrolled 582 adult patients admitted to the respiratory intensive care unit (RICU). We collected the clinical and laboratory data. X-tile software was used to determine the optimal cut-off values for the AGR. Patients were divided into three groups according to the AGR (low AGR group with AGR < 0.8, medium AGR group with AGR ranging from 0.8 to 1.1, and high AGR group with AGR > 1.1). Kaplan-Meier analysis was used for survival analysis. A Cox proportional hazard model was applied to the univariate and multivariate analyses for the potential predictors associated with survival.

**Results:**

Our present study showed that the AGR was related to the 28-day survival of critically ill patients in the RICU. The rate of pneumonia in the low AGR group was significantly higher than that in the other groups. Patients with a lower AGR present an increased risk of 28-day mortality compared to patients with a higher AGR. Cox regression analysis showed that the AGR might be an independent predictor of prognosis to 28-day survival in critically ill patients in the RICU. Medium and high AGR values remained independently associated with better 28-day survival than low AGR values (HR: 0.484 (0.263-0.892) (*p* = 0.02); HR: 0.332 (0.166-0.665) (*p* = 0.002)).

**Conclusion:**

The AGR might be an independent predictor of prognosis in critically ill patients.

## 1. Introduction

Critical illnesses in the intensive care unit (ICU), such as sepsis, could be a global health priority [1, 2]. Being bedridden and the use of some drugs may affect the nutritional status of critically ill patients. Some studies have shown that systemic nutritional status is related to the prognosis of critically ill patients [3].

Serum albumin is commonly used as a surrogate of nutritional status, and serum globulin is cited to assess the severity of chronic inflammation [4]. Hypoalbuminemia has turned out to be associated with critical illness through multiple mechanisms [5]. The albumin-globulin ratio (AGR), calculated as serum albumin/(serum protein–serum albumin), has been reported to be a novel prognosticator of many diseases, such as lung cancer and microscopic polyangiitis [6, 7]. However, previous studies have mainly focused on patients with cancer and some chronic diseases, and thus far, no study has investigated the association of the AGR with the prognosis of critically ill patients. We aimed to conduct this study to determine whether the AGR would predict survival in patients with critical illness.

## 2. Materials and Methods

### 2.1. Participants and Study Design

We conducted a retrospective cohort study of 582 adult patients admitted to the respiratory intensive care unit (RICU) in the College of Pulmonary and Critical Care Medicine, Chinese PLA General Hospital, from 1 July 2008 to 31 December 2017. We collected the clinical data of these patients for retrospective analysis. The inclusion criteria of the study were as follows: (1) adult patients (age ≥ 18 years old), (2) patients with a laboratory examination of serum albumin and serum total protein, (3) patients with a laboratory examination of liver function, and (4) patients with a RICU stay of 2 to 100 days. The exclusion criteria of the study were as follows: (1) patients younger than 18 years old and (2) patients with insufficient AGR data. The primary outcome of the study was 28-day mortality.

### 2.2. Clinical and Laboratory Data Collection

The baseline data of the patients included were collected during the stay in the RICU. The AGR was calculated by the following formula: AGR = serum albumin/(serum protein − serum albumin) [8].

### 2.3. Statistical Analysis

*X*-tile 3.6.1 software (Yale University, New Haven, CT, USA) was used to determine the optimal cut-off values for the AGR. IBM SPSS Statistics 21.0 software (SPSS Inc., Chicago, IL, USA) was applied to complete the statistical analysis. Data are described as numbers (percentages) for categorical variables, means ± standard deviations for normally distributed variables, and medians (interquartile ranges) for skewed distributed variables. Patients were divided into three groups according to the AGR. *Χ*^2^ tests were used to analyze the relationship between the clinical parameters and AGR. Kaplan-Meier analysis was used for survival analysis. A Cox proportional hazard model was applied to the univariate and multivariate analyses for the potential predictors associated with survival. The results were considered statistically significant with a two-sided *p* value <0.05.

## 3. Results

### 3.1. Clinical Characteristics of Patients

The cohort included 198 females and 384 males, with an average age of 62 years old (range 18-98 years old). The AGR of these patients ranged from 0.2 to 2.24, with a median value of 1.06. Data on liver function-related factors, such as alanine aminotransferase (ALT), aspartate aminotransferase (AST), total bilirubin (TBIL), and direct bilirubin (DBIL), were collected. The average ALT, AST, TBIL, and DBIL levels were 34 U/L, 37 U/L, 14.8 *μ*mol/L, and 6.8 *μ*mol/L, respectively. Among these 582 patients, 267 (45.9%) patients had a comorbidity of pneumonia, and 93 (15.8%) patients had a comorbidity of respiratory failure. The average length of stay (LOS) in the RICU was 16 days. The 28-day mortality for the entire cohort was 10.0% (Table 1).

### 3.2. Identification of the Optimal Cut-off Value for AGR

**Figure 1:**
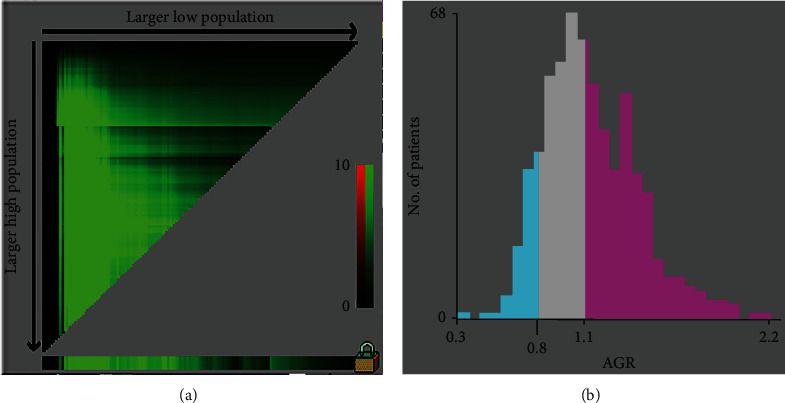
*X*-tile analyses of 28-day mortality performed by using patients' data to determine the optimal cut-off values for *D*-dimer. The sample of critically ill patients was equally divided into training and validation sets. *X*-tile plots of training sets are shown in (a), with plots of matched validation sets shown in the smaller inset (a). The optimal cut-off value highlighted by the black circles in (a) is shown in histograms of the entire cohort (b).

We used *X*-tile software to determine the optimal cut-off value for the AGR of 28-day mortality. *X*-tile is a robust graphical tool verified by Yale University [9], and the cut-off value of the AGR by *X*-tile could be convincing. The optimal cut-off values determined by *X*-tile for the AGR were 0.8 and 1.1 (Figure 1). According to the cut-off value, we divided the patients into three groups: low AGR group with AGR < 0.8, medium AGR group with AGR ranging from 0.8 to 1.1, and high AGR group with AGR > 1.1. Ninety-four patients were in the low AGR group, 237 patients were in the medium AGR group, and 251 patients were in the high AGR group. For the patients with pneumonia, there were 58 (61.7%), 114 (48.1%), and 95 (37.8%) patients in the low AGR, medium AGR, and high AGR groups, respectively (*p* < 0.001) (Table 1). The rate of patients with pneumonia in the low AGR group was significantly higher than that in the other groups. The 28-day mortality of the low AGR, medium AGR, and high AGR groups was 19.1%, 10.5%, and 6.0%, respectively (*p* = 0.001).

### 3.3. Kaplan-Meier Analysis

**Figure 2:**
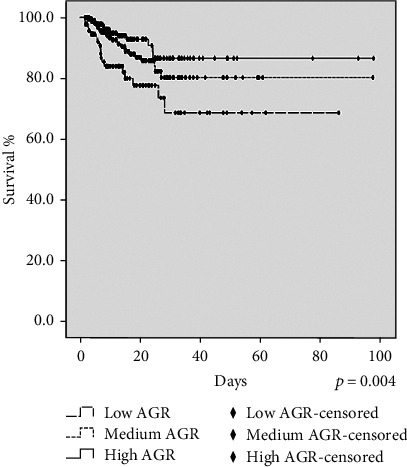
Kaplan-Meier analysis showed that the low AGR group had the worst 28-day survival, and the high AGR group had the best 28-day survival.

Figure 2 illustrates the Kaplan-Meier analysis of 28-day survival with log − rank *p* = 0.004. Kaplan-Meier analysis showed that the low AGR group had the worst 28-day survival, and the high AGR group had the best 28-day survival, indicating that the AGR might be related to the prognosis of critically ill patients in the RICU.

### 3.4. Univariate and Multivariate Analyses of Predictive Factors for 28-Day Survival

Cox proportional hazard regression analysis was conducted to determine the potential prognostic factors for the 28-day survival of critically ill patients in the RICU. The univariate analysis results showed that the medium AGR and high AGR groups had better 28-day survival than the low AGR group, with hazard ratios (HRs) of 0.523 (0.285-0.959) (*p* = 0.036) and 0.330 (0.166-0.655) (*p* = 0.002), respectively. Meanwhile, elevated DBIL and TBIL and age ≥ 65 years old indicated a correlation with worse 28-day survival (HR: 2.232 (1.306-3.815) (*p* = 0.003), HR: 2.016 (1.156-3.517) (*p* = 0.014), and HR: 2.19 (1.231-3.896) (*p* = 0.008)), respectively). In the multivariate analysis, medium and high AGR values remained independently associated with better 28-day survival (HR: 0.484 (0.263-0.892) (*p* = 0.02) and HR: 0.332 (0.166-0.665) (*p* = 0.002)) than low AGR values. Age exceeding 65 years remained an independent factor for worse 28-day survival (HR: 2.196 (1.23-3.92) (*p* = 0.009)) (Table 2).

## 4. Discussion

To the best of our knowledge, this is the first study to examine the prognostic value of the AGR in terms of 28-day survival in critically ill patients. Our present study showed that the AGR was related to the 28-day survival of critically ill patients in the RICU. The rate of pneumonia in the low AGR group was significantly higher than that in the other groups. Patients with a lower AGR present an increased risk of 28-day mortality compared to patients with a higher AGR. Cox regression analysis showed that the AGR might be an independent predictor of prognosis to 28-day survival in critically ill patients in the RICU.

The condition of patients in the ICU is usually poor, causing a serious economic burden to society and family. Therefore, it is very important to find novel markers in the prognosis of critical illness, which might be helpful in targeted therapy. Agnello et al. showed that monocyte distribution width was associated with sepsis in the ICU [10]. In addition, midregional proadrenomedullin (MR-proADM) has also shown a predictive role in the mortality of critically ill patients in the ICU [11].

The nutritional status of critically ill patients will be affected due to long-term bed stays and inflammation. Therefore, nutritional status was associated with the prognosis of critically ill patients. Nutritional risk has been proven to be a predictor of 28-day and ICU mortality [12]. Many studies have focused on nutrition status and its predictable role in critically ill patients, as well as nutrition therapy [13, 14]. Albumin is commonly regarded as a biological marker for assessing nutritional status. Critical illness can change the distribution of intravascular and extravascular albumin. In addition, the rate of albumin synthesis and degradation will also be affected, and cytokines, such as tumor necrosis factor-*α* (TNF-*α*) and interleukin-6 (IL-6), turned out to be responsible for the altered albumin in critically ill patients [5]. The etiologies of hypoalbuminemia include malnourishment, hepatic impairment, and decreased hepatic synthesis of albumin [4].

The AGR has drawn increasing attention for its potential role as a predictor in many diseases. The AGR was assumed to be correlated with prognosis in colorectal cancer [15], lung cancer [16], gliomas [17], and renal cell carcinoma [18]. On the one hand, the AGR reflects the nutritional status of the patients, which might indicate the prognosis of the disease. On the other hand, the AGR might be associated with inflammation. Albumin is related to many inflammatory markers, such as C-reactive protein (CRP) [19], interleukin-1(IL-1), IL-6, and TNF-*α* [4]. Undurti proposed that albumin mobilizes polyunsaturated fatty acids (PUFAs) from the liver and aids in the formation of cytoprotective bioactive lipids, such as lipoxins (LXs), resolvins, and protectins that may underlie its benefits [20]. In our present study, the rate of patients with pneumonia in the low AGR group was significantly higher than that in the other groups, which might indicate that the AGR correlated with pneumonia in critically ill patients. The mechanism of the AGR and critically ill diseases, such as pneumonia, needs to be researched in the future.

Our study showed that the AGR might be an independent predictor of the prognosis to 28-day survival in critically ill patients. A lower AGR indicated a worse prognosis in critically ill patients. We might find an efficient way to increase albumin and the AGR; so, we could give critically ill patients an encouraging prognosis.

## 5. Limitations

There are some limitations to our retrospective study. First, this is a single-centre retrospective study, and the sample size is relatively small compared to that in a multicentre study. In addition, there might be selection bias, which is the defect in most single-centre retrospective studies. Second, we did not collect the AGR during the whole stay in the RICU, and we cannot accurately analyze the trend of the AGR with disease progression. Finally, our study was a clinical study and did not examine the exact mechanism by which the AGR affects the prognosis of critically ill patients. More studies of the mechanisms of the AGR and disease are needed in the future.

## 6. Conclusions

In the present study, we showed that the AGR was correlated with the 28-day survival of critically ill patients in the RICU. Patients with a lower AGR present an increased risk of 28-day mortality compared to patients with a higher AGR. AGR might be an independent predictor of prognosis to 28-day survival in critically ill patients. Multi-institutional studies are needed to confirm our present findings. How to adjust the therapeutic strategies of critically ill patients with a poor prognosis determined by the AGR might be a subject worth studying in the future.

## Figures and Tables

**Table 1:** The clinical characteristics of patients admitted to RICU by AGR.

Variable	Total	Low AGR (≤0.8)	Medium AGR (0.8-1.1)	High AGR (>0.8)	*p* value
Demographics					
Gender (female, *n*,%)	198 (34.0%)	26 (27.7%)	86 (36.3%)	86 (34.3%)	0.319
Age (y)	62	61	63	61	0.196
Comorbidity					
Pneumonia (*n*, %)	267 (45.9%)	58 (61.7%)	114 (48.1%)	95 (37.8%)	<0.001
Respiratory failure (n,%)	93 (15.8%)	16 (17.0%)	44 (18.6%)	33 (13.1%)	0.273
Laboratory variables					
ALT ave (U/L)	34	34	38	29	0.107
AST ave (U/L)	37	52	39	30	0.047
DBIL ave (*μ*mol/L)	6.8	6.6	7	6.6	0.967
TBIL ave (*μ*mol/L)	14.8	13.5	15.1	14.9	0.828
Patient outcomes					
28-day mortality	10.0%	19.1%	10.5%	6.0%	0.001
Length of stay days	16	17	17	15	0.214

**Table 2:** Univariate and multivariate analysis of prognostic factors for 28-day survival in critically ill patients.

Variable	Univariate analysis		Multivariate analysis	
	HR (95% CI)	*p* value	HR (95% CI)	*p* value
Low AGR				
Medium AGR	0.523 (0.285-0.959)	0.036	0.484 (0.263-0.892)	0.02
High AGR	0.330 (0.166-0.655)	0.002	0.332 (0.166-0.665)	0.002
Age (≥65 y vs. <65 y)	2.19 (1.231-3.896)	0.008	2.196 (1.23-3.92)	0.009
Gender (female vs. male)	0.839 (0.481-1.464)	NS		
AST (elevated vs. normal)	1.408 (0.799-2.481)	NS		
ALT (elevated vs. normal)	1.202 (0.659-2.194)	NS		
DBIL (elevated vs. normal)	2.232 (1.306-3.815)	0.003	1.65 (0.734-3.711)	NS
TBIL (elevated vs. normal)	2.016 (1.156-3.517)	0.014	1.513 (0.648-3.529)	NS

Abbreviation: AGR: Albumin-to-globulin ratio; HR: hazard ratio; AST: aspartate aminotransferase; ALT: alanine aminotransferase; DBIL:direct bilirubin; TBIL: total bilirubin; NS: not significant.

## Data Availability

The data used to support the findings of this study are available from the corresponding author upon request.
